# Pre-lacteal feeding practices, determinants, and early health outcomes among children under 2 years of age in Nogob Zone, Somali Region, Ethiopia: a facility-based cross-sectional study

**DOI:** 10.1080/16549716.2026.2643073

**Published:** 2026-03-16

**Authors:** Addisu Assfaw Ayen, Wali Ahmed Nur, Musse Ahmed Ibrahim, Mohamed Ayanle Hassan, Tsion Gurju Awgichew, Mohamed Mahdi Hussen, Aidrose Ahmed Mohamud, Mulugeta Ashagrie Bekahagn, Habtamu Muluken Mekonen, Kalid Fuad Sheikmusse, Abdihalim Abdulahi Mohamed, Getachew Worku Melese, Ahmednur Iman Gamadid, Fisseha Nigussie Dagnew, Getachew Yitayew Tarekegn

**Affiliations:** a Department of Internal Medicine, College of Health Sciences, Debre Tabor University, Debre Tabor, Ethiopia; b Department of Public Health, Garbo Primary Hospital, Somali Region, Ethiopia; c Department of General Surgery, Jigjiga University, Jigjiga, Ethiopia; d Department of Planning and Management, Somali Regional Health Bureau, Jigjiga, Ethiopia; e Department of General Medicine, Bahir Dar University, Bahir Dar, Ethiopia; f Department of Nursing, Garbo Primary Hospital, Somali Region, Ethiopia; g Department of Public Health, Somali Regional Health Bureau, Jigjiga, Ethiopia; h Department of Pediatrics and Childhood Health, Debre Tabor University, Debre Tabor, Ethiopia; i Department of Public Health, Jigjiga University, Jigjiga, Ethiopia; j Department of Nursing, Jigjiga University, Jigjiga, Ethiopia; k Department of Medicine, Garbo Primary Hospital, Garbo, Ethiopia; l Clinical Pharmacy Unit, Department of Pharmacy, College of Health Sciences, Debre Tabor University, Debre Tabor, Ethiopia

**Keywords:** Pre-lacteal feeding, neonatal health, breastfeeding initiation, determinants, pastoralist communities

## Abstract

**Background:**

Pre-lacteal feeding, the provision of foods or liquids other than breast milk before breastfeeding, undermines optimal infant feeding and increases neonatal morbidity. Evidence from pastoralist settings in Ethiopia is limited.

**Objective:**

This study aimed to assess the prevalence, determinants, and early health outcomes of pre-lacteal feeding among children aged 2 years in the Nogob Zone, Somali Region, Ethiopia.

**Methods:**

A facility-based cross-sectional study was conducted from June to December 2025 among 609 mother – infant pairs attending seven health centers and one primary hospital. Stratified and systematic sampling were applied. Data were collected via structured questionnaires and analyzed using multivariate logistic regression. Adjusted odds ratios with 95% confidence intervals identified determinants and associated neonatal outcomes.

**Results:**

Pre-lacteal feeding prevalence was 40.1% (95% CI: 36.2%–44.0%), with plain water, sugar water, and animal milk being the most common. Determinants included rural residence (AOR = 2.05; 95% CI: 1.30–3.22), no antenatal care (AOR = 2.10; 95% CI: 1.28–3.45), birth order ≥5 (AOR = 2.05; 95% CI: 1.25–3.35), maternal illiteracy (AOR = 2.90; 95% CI: 1.65–5.10), and cesarean delivery (AOR = 4.55; 95% CI: 1.72–12.10). Pre-lacteal feeding increased the odds of neonatal complications (AOR = 2.30; 95% CI: 1.35–3.95).

**Conclusions:**

Pre-lacteal feeding is common in the Nogob zone and is linked to adverse neonatal outcomes. Maternal education, antenatal care promotion, early breastfeeding initiation, and culturally sensitive community interventions are essential to reduce pre-lacteal feeding and improve neonatal health in pastoralist settings.

## PAPER CONTEXT

**Main findings:** Pre-lacteal feeding practices are common in the study area and are significantly influenced by maternal, household, and healthcare factors.

**Added knowledge:** This study identifies key determinants of early infant feeding practices and their association with neonatal health outcomes in pastoralist and resource-limited settings.

**Global health impact for policy and action:** Findings highlight the need for targeted interventions and health policies to promote optimal breastfeeding practices and reduce neonatal morbidity in similar low-resource regions.

## Background

Optimalinfant feeding is vital for child survival and health outcomes. Exclusive breastfeeding for the first six months provides crucial nutrients and immune protection against diseases like diarrhea and pneumonia, yet only 44% ofinfants globally are exclusively breastfed [1]. Pre-lacteal feeding, common especially in low- and middle-income countries, hampers early breastfeeding by delaying its initiation and reducing exclusivity, leading to higher infection risks. Prevalence rates of pre-lactealfeeding vary, ranging from 10% to 75% in low-resource areas, influenced by sociocultural and health system factors, such as maternal education and access to healthcare [2,3]. These elements underscore the need to address barriers to optimal breastfeeding practices worldwide. 

In Ethiopia, PLF is a significant public health concern. Studies report prevalence from 8% to over 40%, dependingon region and study population. Common pre-lacteal feeds include water, sugarwater, butter, and animal milk, often influenced by cultural beliefs [4–6]. Key determinants for this practice include rural residence, limited healthcare access, inadequate antenatal care, delayed breastfeeding initiation, and lack of infant feeding counseling. Furthermore, socioeconomic factors and maternal age also play a role. Despite government initiatives advocating for exclusive breastfeeding, many infants inunderserved communities remain vulnerable due to entrenched cultural norms and poor health service utilization, underscoring the need for tailored interventions to overcome these barriers [4,7,8].

This study in the Nogob Zone, Somali Region, builds on previous research on infant feeding practices in Ethiopia by integrating sociodemographic, service-related, and behavioral factors. It specifically investigates how antenatal care, maternal literacy, and mode of delivery affect pre-lacteal feeding practices, addressing gaps in earlier analyses that primarily focused on urban/rural ornational-level populations without exploring underlying behavioral mechanisms. Research highlights factors such as antenatal care and maternal literacy butfails to consider the sociocultural and geographic contexts affecting feeding behaviors in nomadic populations [5,9]. This study critiques pre-lacteal feeding (PLF) practices in the Somali Region’s Nogob Zone, linking PLF to malnutrition and neonatal risks. It provides critical insights into child health outcomes through a multi-site approach aimed at enhancing findings’ relevance for other pastoralist populations. The research proposes community interventions to reduce PLF and improve health inresource-limited settings [9,10]. This work contributes essential evidence to the public health discourse on neonatal and child survival in the region.

Emerging evidence from other low-resource and pastoralist settings highlights that pre-lacteal feeding (PLF) is not only a localized Ethiopian phenomenon but also a widespread challenge. Recent studies from sub-Saharan Africa, Asia, and the Middle East (2020–2025) demonstrate similar sociocultural, geographic, and health system determinants of PLF, emphasizing the broader relevance of understanding and addressing these practices [12–14]. By situating the Somali Region findings within this global discourse, the study underscores the potential for cross-context learning and the design of interventions applicable beyond Ethiopia.

This study aims to estimate the prevalence of pre-lacteal feeding in the Nogob Zone, Somali Region, and to identify its sociodemographic, obstetric, and health service-related determinants. It also examines the association between pre-lacteal feeding and adverse neonatal health outcomes, including infections and feeding difficulties. The findings will provide context-specific evidence to inform public health strategies, promote timely initiation of breastfeeding, and improve neonatal health in underserved communities.

The Nogob Zone, located in the Somali Region of Ethiopia, is predominantly pastoralist, with limited access to healthcare, high rates of childmalnutrition, and strong cultural influences on infant feeding practices. Understanding these sociocultural norms and health system constraints is essential, as they shape pre-lacteal feeding behaviors and neonatal outcomes. Evidence from this context can guide targeted interventions to promote optimal breastfeeding practices in similar resource-limited pastoralist settings.

## Methods

### Study design, period, and setting

A facility-based cross-sectional study in the Nogob Zone of the Somali Region, Ethiopia, occurred from 21 June to 21 December 2025. The zone, inhabited by Somali pastoralist communities, has limited health infrastructure, including one hospital and 13 health centers. Data were collected from Garbo Primary Hospital and eight health centers, with distances ranging from 13 km to 155 km from the hospital. The population of Garbo is 127,000, contributing to a total of approximately 400,000 in the Nogob Zone. The study adhered to the STROBE guidelines for cross-sectional studies [15].

### Study population and eligibility criteria

The facility-based cross-sectional study focused on mothers or primary caregivers of infants aged 0–24 months attending health services in the Nogob Zone. Inclusion criteria required participants to be 18 years or older, the primary caregiver since birth, and capable of providing reliable feeding information with consent. Exclusion criteria eliminated caregivers that were critically ill, not the primary caregiver since birth, or had incomplete data. These eligibility criteria were strictly applied to enhance the study’s validity and transparency, ensuring accurate representation of the target population and comparability of data.

### Health facility selection

The study in the Nogob Zone, Somali Region, involved purposively selected health facilities to ensure geographic representativeness and included both high- and low-volume centers offering maternal and child health services. Selection criteria focused on accessibility, availability of postnatal and immunization services, and capacity for routine neonatal care. This method enabled thorough data collection from various facilities and showcased healthcare access patterns among pastoralist communities, enhancing transparency in the study’s context and findings.

### Sampling frame and attendance records

The study constructed a sampling frame from attendance records of maternal and child health services, including logs from nurses and health extension workers. These records detailed mothers attending antenatal, postnatal, and immunization services, capturing relevant data such as age, parity, residence, and child’s age to identify eligible participants. This systematic review enhanced transparency and accountability in the selection process.

### Sample size determination

The required sample size was determined using the single population proportion formula, which is suitable for estimating population prevalence with a defined level of precision and confidence [16].

*N* = Z^2^×P(1-P)/d^2^

Where:

- where n is the minimum required sample size for a simple random sample;
- Z is the standard normal deviation corresponding to the desired confidence level—1.96 for 95% confidence;
- P is the anticipated prevalence of the outcome of interest (pre-lacteal feeding).
- Disclose the margin of error (precision) desired around the prevalence estimate.

Where: Zα/_2_ = for a 95% confidence level, *p* = 0.40, the estimated prevalence of pre-lacteal feeding in the Somali Region of Ethiopia based on previous studies, and d = 0.05, the margin of error.

Substituting the values:

*n* = (1.96)^2^ ×0.40 × 0.60(0.05)_2_ = 3.84 × 0.240.0025 = 369

Since the study employed multistage sampling, a design effect of 1.5 was applied:

*n* = 369 × 1.5 = 554

To account for potential non-response (10%), the final sample size was adjusted as follows:

N final = 554+(0.10 × 554) =609. Therefore, the final sample size for this study was 609 mother-infant pairs.

### Sampling procedure

A facility-based stratified sampling approach was implemented in the Nogob Zone, targeting mother-infant pairs across seven health centers and one primary hospital. Based on previous attendance records, the final sample consisted of 609 participants, with a larger initial estimate of 641 to account for non-responses. Sample sizes were proportionally allocated to each facility using a specific formula related to eligible infants (Table 1). Systematic sampling from attendance lists was utilized, with random selection of infants when necessary, ensuring proportional representation within the final sample.

**Table 1.** Distribution of study participants across health facilities in Nogob Zone, Somali Region, Ethiopia.

Health Facility (Stratum)	Estimated sample size (n)*	Actual sample size (n)
Maleyko Health Center	92	85
Dardheer Health Center	80	75
Dhuxun Health Center	69	64
Segeg Health Center	57	56
Eleuni Health Center	69	64
Gerbo Health Center	46	44
Hareri Health Center	56	52
Garbo Primary Hospital	172	169
Total	641	609

Allocated Sample refers to the number of participants planned for recruitment at each facility based onprevious attendance records. ‘Actual Recruited Sample’ refers to the number of participants successfully enrolled in the study.

The total sample sizewas proportionally allocated to the study sites according totheir catchment population size (‘Allocated Sample’). The number of participants actually enrolled at each site is reported as ‘Actual Recruited Sample’.

### Data collection instrument and procedures

Data were collected using a structured checklist adapted from validated surveys on infant feeding and maternal health. The instrument, informed by tools like the Ethiopian Demographic and Health Survey, assessed sociodemographic characteristics, breastfeeding practices, maternal and service-related factors, and infant health outcomes [17,18]. Initially developed in English, it was translated into Somali and back-translated to ensure consistency. Prior to data collection, the questionnaire was pre-tested for clarity and cultural appropriateness [19]. Trained data collectors conducted face-to-face interviews, supported by daily supervision to ensure data quality and completeness. Information on child infections and respiratory conditions was obtained through the maternal report during structured interviews. Mothers were asked whether their child experienced symptoms such as fever, cough, difficulty breathing, or lethargy. These outcomes are described as maternally reported symptoms of infection or respiratory illness and were not clinically verified.

### Study variables

In this study, several variables were collected to examine the prevalence, determinants, and outcomes of pre-lacteal feeding. The primary exposure variable was pre-lacteal feeding, defined as giving the newborn any food or drink other than breast milk before initiating breastfeeding. The outcome variables included neonatal complications, such as infections (sepsis, pneumonia, diarrhea), feeding difficulties, and jaundice, as well as neonatal survival status at 28 days of life. Sociodemographic variables included maternal and paternal age, education, occupation, household income, household size, and place of residence. Obstetric and child-related factors included parity, birth order, birth interval, gestational age at birth, mode and place of delivery, skilled birth attendance, antenatal care visits, birth weight, and sex of the newborn. Health service – related variables included postnatal care attendance, counseling on breastfeeding, type of health facility, and distance to the nearest facility. Additionally, cultural and behavioral factors, such as maternal knowledge, beliefs, and family or community influences on infant feeding practices, were considered. These variables were measured through structured interviews with mothers, review of health records, and standardized anthropometric and clinical assessments, ensuring consistency and reliability for subsequent analyses.

#### Variable definitions and measurements

- Pre-lacteal feeding: receipt of any food or liquid other than breast milk before breastfeeding initiation within the first 3 days of life.
- Early initiation of breastfeeding: breastfeeding initiated within 1 hour postpartum.
- Low birth weight: <2500 grams.
- Preterm birth: gestational age <37 completed weeks.
- **Early complications:** Defined as maternally reported illnesses or symptoms within the first 28 days of life, such as fever, breathing difficulty, poor feeding, or hospital admission. Data were gathered through caregiver recall in interviews, excluding any illnesses after the neonatal period from this analysis.

All variables were defined based on standard epidemiological criteria to enhance comparability with existing studies.

#### Data quality assurance

To maintain data integrity and accuracy, various quality assurance measures were implemented throughout the research. A structured questionnaire, adapted from validated instruments in maternal and child health, underwent rigorous translation from English to Somali and back to ensure semantic equivalence. A pre-test among caregivers refined question clarity and cultural relevance. Data collectors, trained in interview techniques and ethical conduct, received daily supervision to ensure adherence to protocols. Post-collection, questionnaires were reviewed for completeness and consistency, focusing on key variables and response plausibility. These processes are vital in enhancing data reliability and align with established health research methodologies for quality assessment.

#### Missing data handling

In accordance with established guidelines for observational studies, the initial step evaluates and categorizes the extent of missing data for key variables. For variables with negligible missingness (<5%), complete-case analysis (listwise deletion) was performed, allowing for unbiased estimates under the assumption of missing completely at random (MCAR). In instances of more significant missing data, and where the missing at random assumption was plausible, multiple imputation with chained equations was used to mitigate bias and precision loss. This method generates multiple complete datasets to reflect the uncertainty in imputed values, with results combined using Rubin’s rules [20]. Sensitivity analyses were conducted to assess the robustness of findings by comparing results from complete-case and imputed datasets, following best practices for missing data assessment.

#### Statistical analysis

Data were processed using EpiData 4.7.0 and SPSS 27, starting with descriptive statistics to summarize participant characteristics and outcomes. Continuous variables were summarized with means and standard deviations, whereas categorical variables used frequencies and percentages. Bivariate logistic regression analyses identified factors associated with pre-lacteal feeding and infant health; variables with *p*-values <0.25 were candidates for multivariate modeling to reduce the risk of omitting important confounders. Multivariate logistic regression analysis was performed to identify factors associated with pre-lacteal feeding, and adjusted odds ratios with 95% confidence intervals were reported. Model specification and interpretation followed standard recommendations for logistic regression. The analyses accounted for the multistage sampling design to ensure valid inferences using survey-adjusted logistic regression. Statistical significance was set at *p* < 0.05, with results presented in tables showing both unadjusted and adjusted estimates.

## Results

### Sociodemographic characteristics of the study participants

A study involving 609 children, with a male predominance of 52.5%, reported a mean age of 4.3 months. Almost half of the mothers (46.0%) were illiterate, and most were housewives (54.2%). Most fathers had at least primary education, with 21.2% achieving college-level education. The majority lived in rural areas (70.4%) and came from married households (88.7%). The average monthly household income was 4300 ETB ± 1,250 [Table 2]

**Table 2.** Sociodemographic characteristics of children and their mothers/caregivers (*N* = 609).

Variable	Category	Frequency (n)	Percentage (%)
Child sex	Male	320	52.5
	Female	289	47.5
Child age (months)	Mean ± SD	4.3 ± 1.6	–
Birth order	1st	150	24.6
	2nd	140	23.0
	3rd	120	19.7
	4th	100	16.4
	≥5th	99	16.3
Birth weight (grams)	Mean ± SD	3180 ± 460	–
Mother’s age at delivery	Mean ± SD	27.5 ± 5.7	–
Mother’s education	Unable to read/write	280	46.0
	Able to read/write	75	12.3
	Primary	90	14.8
	Secondary	90	14.8
	College and above	74	12.1
Mother’s occupation	Housewife	330	54.2
	Farmer	120	19.7
	Merchant	50	8.2
	Civil servant	40	6.6
	Other	69	11.3
Father’s education	Unable to read/write	250	41.0
	Able to read/write	60	9.8
	Primary	80	13.1
	Secondary	90	14.8
	College and above	129	21.2
Place of residence	Urban	180	29.6
	Rural	429	70.4
Marital status	Married	540	88.7
	Single	20	3.3
	Divorced	25	4.1
	Widowed	24	3.9
Monthly household income	Mean ± SD	4,300 ± 1,250	–

^a^SD = standard deviation.

^b^Percentages may not sum to 100because of rounding.

^c^Birth order refers to the chronologicalposition of the child in the family.

^d^Household income is reported in the Ethiopian Birr (ETB).

^e^Ableto read/write indicates basic literacy without formal education.

### Breastfeeding practices and pre-lacteal feeding

Most children (95.2%) were breastfed, with 62.4% initiating breastfeeding within the first hour of birth. Colostrum was given to 75.5% of infants, while 40.1% of children experienced pre-lacteal feeding, primarily with plain water (32.8%), sugar water (24.6%), or animal milk (14.3%), typically for one day [Table 3].

**Table 3.** Breastfeeding and pre-lacteal feeding practices (*N* = 609).

Variable	Category	Frequency (n)	Percentage (%)
Ever breastfed	Yes	580	95.2
	No	29	4.8
Breastfeeding initiated within 1 hour	Yes	380	62.4
	No	229	37.6
Colostrum given	Yes	460	75.5
	No	149	24.5
Pre-lacteal feeding is given.	Yes	244	40.1
	No	365	59.9
Type of pre-lacteal feed	Plain water	80	32.8
	Sugar water	60	24.6
	Honey	30	12.3
	Animal milk	35	14.3
	Infant formula	15	6.1
	Butter/ghee	5	2.0
	Herbal preparation	10	4.1
Duration of pre-lacteal feeding	Single time	80	32.8
	1 day	70	28.7
	1–3 days	50	20.5
	>3 days	44	18.0

^a^Colostrum refers to the first thick, yellowish breast milk produced within 24 hours of birth.^b^Pre-lacteal feeding includes any food or liquid given before initiating breastfeeding.^c^‘Single time’ indicates that the pre-lacteal feed was given only once immediately after birth.^d^Percentages may not sum to 100 because of rounding or multiple types of pre-lacteal feeds reported.

### Child health outcomes

Child survival was notably high, with 98.7% of children alive during data collection. Mothers indicated complications in 20.0% of cases, mainly infections or sepsis (31.1%), diarrhea or vomiting (25.4%), and aspiration or pneumonia (12.3%). Other reported issues included feeding difficulties, jaundice, allergic reactions, delayed milk supply, and hypothermia. Additionally, 12% of children showed signs suggestive of infection, while 8% exhibited respiratory symptoms potentially indicating pneumonia [Table 4].

**Table 4.** Early health outcomes of children (*N* = 609).

Outcome	Category	Frequency (n)	Percentage (%)
Child alive	Yes	601	98.7
	No	8	1.3
Child developed complications.	Yes	122	20.0
	No	487	80.0
Types of complications (*n* = 122)	Infection/sepsis	38	31.1
	Diarrhea/vomiting	31	25.4
	Aspiration/pneumonia	15	12.3
	The allergic reaction	8	6.6
	Feeding problems	10	8.2
	Jaundice	10	8.2
	Hypothermia	3	2.5
	Delayed milk supply	7	5.7
	Other	0	0.0

^a^Percentages for complications were calculated among children who developed any complication (n = 122). ^b^Neonatal complications refer to maternally reported symptoms occurring within the first 28 days of life. ^c^Neonatal complications were collected via maternal recall and were not clinically verified. This category includes fever, difficulty breathing, lethargy, or other symptoms reported in the first 28 days of life.

### Factors associated with pre-lacteal feeding

Multivariate logistic regression indicated multiple independent predictors of pre-lacteal feeding. Children of mothers who cannot read or write had almost three times the odds of receiving such feeds compared to those with mothers having college education (AOR = 2.90; 95% CI: 1.65–5.10; *p* < 0.001). Those born to literate but uneducated mothers were also at risk (AOR = 2.05; 95% CI: 1.05–3.95; *p* = 0.03). Additional significant factors included rural residence (AOR = 2.05; 95% CI: 1.30–3.22; *p* = 0.002), higher birth order (≥5th) (AOR = 2.05; 95% CI: 1.25–3.35; *p* = 0.004), non-attendance of antenatal care (AOR = 2.10; 95% CI: 1.28–3.45; *p* = 0.003), and cesarean delivery was associated with PLF (AOR = 4.55; 95% CI: 1.72–12.10), although the small number of cesarean cases (*n* = 19) limits the precision of this estimate. [Table 5]

**Table 5.** Multivariate logistic regression for pre-lacteal feeding (*N* = 609).

Variable	Category	Pre-lacteal feeding n (%)	COR (95% CI)	AOR (95% CI)	*p*-value
Child sex	Male	160 (52.0)	1.12 (0.79–1.58)	1.09 (0.76–1.56)	0.64
	Female	149 (48.0)	Ref	Ref	–
Birth order	1st	41 (16.8)	Ref	Ref	–
	2nd	35 (14.3)	1.22 (0.72–2.08)	1.18 (0.69–2.02)	0.55
	3rd	45 (18.4)	1.45 (0.87–2.42)	1.34 (0.81–2.22)	0.25
	4th	32 (13.1)	1.56 (0.91–2.69)	1.44 (0.83–2.50)	0.20
	≥5th	91 (37.3)	2.28 (1.37–3.80)	2.05 (1.25–3.35)	0.004
Maternal education	Unable to read/write	150 (61.5)	3.20 (1.87–5.47)	2.90 (1.65–5.10)	<0.001
	Able to read/write	45 (18.5)	2.10 (1.12–3.95)	2.05 (1.05–3.95)	0.03
	Primary	20 (8.2)	1.50 (0.63–3.55)	1.48 (0.62–3.50)	0.37
	Secondary	20 (8.2)	1.00 (0.47–2.12)	0.96 (0.44–2.08)	0.92
	College+	9 (3.7)	Ref	Ref	–
Residence	Urban	110 (45.1)	Ref	Ref	–
	Rural	139 (54.9)	2.32 (1.55–3.50)	2.05 (1.30–3.22)	0.002
ANC attendance	Yes	190 (77.9)	Ref	Ref	–
	No	54 (22.1)	2.40 (1.52–3.82)	2.10 (1.28–3.45)	0.003
Mode of delivery	Vaginal	225 (92.2)	Ref	Ref	–
	C-section	19 (7.8)	4.90 (1.88–12.80)	4.55 (1.72–12.10)	0.003

^a^AOR = adjusted odds ratio; COR = crude odds ratio; CI = confidence interval. ^b^Ref = reference category. ^c^Statistically significant variables are indicated with (*p* < 0.05).^d^AOR estimates for subgroups with small sample sizes (e.g. cesarean deliveries) may be unstable.

### Factors associated with child complications

Pre-lacteal feeding significantly increases the risk of complications (AOR = 2.30; 95% CI: 1.35–3.95; *p* = 0.002). Low birth weight (<2500 g) (AOR = 3.20; 95% CI: 1.60–6.40; *p* = 0.001) and preterm birth (AOR = 2.80; 95% CI: 1.45–5.40; *p* = 0.002) are also key predictors. Delayed initiation of breastfeeding (≥1 hour) correlates with greater odds of complications, though not statistically significant (AOR = 1.60; 95% CI: 0.90–2.85; *p* = 0.11) [Table 6].

**Table 6.** Multivariate logistic regression for child complications (*N* = 122).

Variable	Category	Child complications n (%)	COR (95% CI)	AOR (95% CI)	*p*-value
Pre-lacteal feeding	Yes	60 (24.6)	2.50 (1.50–4.20)	2.30 (1.35–3.95)	0.002*
	No	62 (17.0)	Ref	Ref	–
Breastfeeding initiation	<1 hr	30 (7.9)	Ref	Ref	–
	≥1 hr	92 (40.2)	1.70 (0.95–3.00)	1.60 (0.90–2.85)	0.11
Birth weight	<2500 g	35 (32.1)	3.50 (1.80–6.80)	3.20 (1.60–6.40)	0.001
	≥2500 g	87 (16.2)	Ref	Ref	–
Gestational age	Preterm	25 (34.7)	3.00 (1.60–5.60)	2.80 (1.45–5.40)	0.002
	Term/post-term	97 (16.0)	Ref	Ref	–

^a^OR = odds ratio; CI = confidence interval. ^b^Ref = reference category. ^c^Statistically significant variables are indicated with (*p* < 0.05).

## Discussion

### Principal findings

In a study of 609 mother-infant pairs conducted from June to December 2025, 40.1% of infants received pre-lacteal feeds, which may not apply to all infants in the Nogob Zone due to the study’s facility-based nature. Factors such as maternal illiteracy, rural residence, lack of antenatal care, higher birth order (≥5), and cesarean delivery were linked to pre-lacteal feeding. Neonatal complications occurred in 20% of infants, with pre-lacteal feeding increasing the risk (AOR = 2.30). Additionally, low birth weight (AOR = 3.20) and preterm birth (AOR = 2.80) significantly predicted early neonatal complications. The findings highlight the need for targeted interventions to enhance maternal education, antenatal care, and early breastfeeding support, especially in rural areas.

### Prevalence of pre-lacteal feeding

In the Nogob Zone, 40.1% of infants received pre-lacteal feeds, significantly surpassing Ethiopia’s national estimates of 25.3%–27.0% [8]. Community studies reveal similarly high rates, with 38.8% in Raya Kobo [21] and 47.3% in Degahbour town [22,23]. Conversely, urban areas report much lower rates, such as 11.1% in Woldia, Kobo, and Lalibela [24], and a national figure of 16.3% from 2019 EDHS data [9]. These discrepancies highlight notable regional and rural-urban differences in pre-lacteal feeding practices, influenced by sociocultural norms, maternal education, health service access, and infant feeding counseling. National averages may thus underestimate pre-lacteal feeding in remote communities where traditional practices prevail.

Pre-lacteal feeding is prevalent in many low- and middle-income countries, particularly in sub-Saharan Africa, where the overall prevalence is estimated at 32.2%. Variations exist, with West African regions reporting higher rates compared to others like Malawi (2.5%) [12]. Southeast Asia also shows high prevalence rates, such as Vietnam at 73.3%, while prelacteal feeding remains highly prevalent in parts of South Asia. In rural India, 40.1% of recently delivered women reported giving pre-lacteal feeds to their newborns in a community-based study, reflecting persistent cultural and sociodemographic influences on early infant feeding practices. Pre-lacteal feeding practices are also documented at high levels in Pakistan, where Demographic and Health Survey data indicate that approximately 64.7% of children received pre-lacteal feeds in some regions [25,26]. Conversely, some East African contexts report lower rates, around 12% [27]. This widespread practice varies regionally due to cultural beliefs, maternal education, and access to health services, often underestimating the burden in rural and pastoralist populations where traditional practices persist.

### Determinants and sociodemographic factors

This study highlights maternal education as a critical factor influencing pre-lacteal feeding practices. Mothers without formal education were significantly more likely to practice pre-lacteal feeding than those who attained higher education. This finding is consistent with studies from southwest Ethiopia and national analyses, which show that lower maternal education is strongly associated with increased pre-lacteal feeding practices [4,8]. Similarly, national meta-analyses indicate that urban mothers are substantially less likely to engage in pre-lacteal feeding due to better access to health information and maternal health services (6,23).

The absence of antenatal care (ANC) correlates with higher rates of pre-lacteal feeding, highlighting the importance of ANC in educating expectant mothers about infant feeding. Systematic reviews of Ethiopian data indicate that attending ANC significantly lowers the likelihood of pre-lacteal feeding, whereas fewer ANC visits are associated with increased odds of such feeding practices in Southwest Ethiopian communities [8,28]. This supports global evidence that maternity care contact points are critical opportunities to reinforce early breastfeeding and discourage pre-lacteal feeds [29].

Higher birth order correlates with increased pre-lacteal feeding, indicating that mothers with more prior births may depend on experience or cultural practices over current health advice. While evidence on the role of birth order is mixed, a national unmatched case – control study based on the 2016 Ethiopian Demographic and Health Survey reported that multiparous mothers had significantly higher odds of practicing pre-lacteal feeding than primiparous mothers [30].

Mode of delivery, particularly cesarean section, was associated with higher rates of pre-lacteal feeding in this study, although the small number of cesarean deliveries warrants cautious interpretation. Evidence from multi-site and national analyses in Ethiopia demonstrates that mothers who delivered by cesarean section had significantly higher odds of providing pre-lacteal feeds compared with vaginal delivery (AOR ≈2.47–2.66) [4,8]. Similarly, a community-based study in Southwest Ethiopia reported that cesarean delivery increased the likelihood of pre-lacteal feeding (AOR ≈4.27) [31]. At the broader regional level, a pooled analysis of Demographic and Health Survey data from 22 sub-Saharan African countries also identified cesarean delivery as a significant predictor of pre-lacteal feeding (adjusted OR ≈2.25) [12]. Although cesarean delivery was associated with higher odds of pre-lacteal feeding, the wide confidence interval indicates that this estimate is imprecise, likely due to the small number of cesarean cases in this population. Therefore, the result should be interpreted with caution.

These findings indicate that cesarean delivery disrupts early mother-infant contact and delays the initiation of breastfeeding, thereby increasing the likelihood of pre-lacteal supplementation. While consistent with both national and international evidence, the wide confidence interval observed in this study highlights the need for cautious interpretation and indicates that further research in larger samples is warranted.

### Cultural driver of pre-lacteal feeding

Sociocultural beliefs, maternal education, and access to health services, especially in rural areas, significantly shape the practice of pre-lacteal feeding. Many caregivers consider colostrum harmful and prefer pre-lacteal feeds such as raw butter or water before breastfeeding [32]. In Ethiopia, cultural traditions intensify these beliefs, with pre-lacteal feeding being seen as protective for newborns [33]. Limited antenatal counseling and inadequate infant feeding knowledge further perpetuate these practices, indicating the need for culturally sensitive public health interventions to encourage early breastfeeding and enhance neonatal health outcomes [34].

### Association with neonatal health outcomes

Pre-lacteal feeding was associated with higher odds of neonatal complications, including infections and diarrhea. Introducing non-breast milk feeds in the first days of life may reduce the intake of colostrum, which provides immunoglobulins and other protective factors, potentially increasing susceptibility to early infections [2,3,35]. Low birth weight and preterm birth were also significant predictors of neonatal complications, consistent with previous studies in Ethiopia and other low-resource settings [11]. Delayed initiation of breastfeeding (≥1 hour) showed higher odds of complications, but this was not statistically significant, likely due to an early initiation among most infants. Infants in rural pastoralist communities are at higher risk than urban populations, facing challenges such as lower hygiene standards, limited neonatal care, and delayed illness recognition [4,7].

### Recommendation and policy implications

Based on the study findings, the following recommendations are proposed to reduce pre-lacteal feeding (PLF) and improve neonatal health in pastoralist populations:

1. Community-Led Education: Engage traditional leaders (‘Ugas’) and respected elders to provide culturally appropriate education on the benefits of exclusive breastfeeding and the risks of PLF. Use oral communication and storytelling adapted to nomadic lifestyles.
2. Mobile Health Outreach: Deploy mobile health teams to reach nomadic and home-based mothers, particularly in remote areas where facility access is limited. Include counseling on breastfeeding initiation within the first hour of birth.
3. Targeted Support for Cesarean Deliveries: Provide individualized breastfeeding guidance for mothers undergoing cesarean sections, emphasizing early initiation and avoiding unnecessary pre-lacteal feeds.
4. Integration with Antenatal and Postnatal Services: Incorporate PLF prevention strategies into routine antenatal care and postnatal follow-up, ensuring education reaches mothers regardless of literacy level.
5. Monitoring and Feedback: Implement community-based monitoring systems to track feeding practices and neonatal outcomes, enabling timely interventions and continuous improvement of outreach strategies.

### Strengths and limitations

This study evaluates pre-lacteal feeding practices in the underserved pastoralist Nogob Zone using a large, multi-site sample for better representativeness and subgroup comparison. It assesses factors such as maternal education and antenatal care attendance alongside child health outcomes, providing insights for public health interventions. However, limitations include a cross-sectional design that hampers causal inference, the exclusion of home births, which may underestimate PLF prevalence, potential recall bias, reliance on maternal reporting for neonatal complications, and unstable estimates in small subgroups. Caution is advised when generalizing findings to wider pastoralist or global populations.

### Public health implications

The findings underscore the necessity of increasing antenatal care access and delivering structured breastfeeding counseling, especially in rural and pastoral communities. There is a call for interventions to engage community leaders to challenge cultural norms that promote pre-lacteal feeding. Multiparous mothers need updated guidance to address reliance on past experiences, and mothers with cesarean deliveries require additional support for early breastfeeding initiation. Community-specific interventions are deemed more effective than general campaigns in reducing pre-lacteal feeding and enhancing neonatal health.

### Future research

Longitudinal studies are essential for establishing the causal relationships between pre-lacteal feeding and neonatal morbidity. Qualitative research into sociocultural factors within pastoralist communities will shed light on behavioral influences. Intervention trials focused on community education and facility support are necessary to decrease pre-lacteal feeding and enhance neonatal outcomes. Additionally, comparative studies across different population types, including rural and urban settings, would aid in shaping targeted public health strategies.

## Conclusion

Pre-lacteal feeding is widespread in the Nogob zone and is linked to neonatal complications. Factors contributing to this practice include maternal illiteracy, rural living, lack of antenatal care, higher birth orders, and cesarean deliveries, with pastoralist communities particularly affected. To combat this issue, it is crucial to enhance maternal education, promote antenatal counseling, encourage early breastfeeding, and address cultural feeding practices to mitigate neonatal morbidity in low-resource settings.

## Data Availability

The datasets generated and analyzed during the current study are available from the corresponding author upon reasonable request.

## Preprints

The authors have not posted any preprint of this manuscript.
